# The influence of psychological intervention-assisted cardiac rehabilitation on kinesiophobia in individuals diagnosed with coronary heart disease

**DOI:** 10.3389/fcvm.2025.1561505

**Published:** 2025-05-27

**Authors:** Xiaoping Guo, Wei Li, Jing Sun, Yanzhuo Ma

**Affiliations:** Department of Cardiology, Bethune International Peace Hospital, Shijiazhuang, Hebei, China

**Keywords:** psychological intervention, cardiac rehabilitation, coronary heart disease, kinesiophobia, quality of life

## Abstract

**Objective:**

Kinesiophobia is prevalent among patients with coronary heart disease (CHD). This study aimed to explore whether psychologically-informed cardiac rehabilitation (CR) could positively influence the reduction of kinesiophobia in CHD patients.

**Methods:**

A total of 86 CHD patients, who sought treatment at Bethune International Peace Hospital between June 2022 and June 2023, were selected and divided into two groups: a psychological intervention group comprising 42 patients and a conventional CR group of 44 patients. The conventional CR group received standard CR intervention, whereas the psychological intervention group underwent CR intervention augmented with psychological support. The effectiveness of these interventions was evaluated using the Self-Rating Depression Scale (SDS), the Self-Rating Anxiety Scale (SAS), the Multidimensional Exercise Self-Efficacy Scale (MSES), the TSK-Heart (for assessing kinesiophobia in cardiac patients), and the Short Form Health Survey (SF-36) scores.

**Results:**

Following the intervention, the SDS and SAS scores of the psychological intervention group were higher than those of the conventional CR group. The MSES dimension scores and the total score of the psychological intervention group were elevated in comparison to the CR group. Conversely, the TSK-Heart dimension scores and the total score of the psychological intervention group were diminished relative to the CR group. Additionally, the SF-36 dimension scores and total score of the psychological intervention group surpassed those of the CR group post-intervention.

**Conclusion:**

The integration of psychologically-supported CR into the rehabilitation regimen for CHD patients effectively mitigates negative emotions, enhances self-efficacy, and markedly reduces kinesiophobia, thus significantly improving overall quality of life.

## 1 Introduction

As one of the most lethal diseases globally, coronary heart disease (CHD) not only poses a grave threat to human life and well-being but also imposes a substantial economic burden on individuals, healthcare systems, and nations worldwide (1). While the incidence and mortality rates of CHD-related conditions have been on the decline in developed countries, they are on the ascent in developing nations (2). Despite the critical role of various therapeutic interventions, including percutaneous coronary intervention (PCI), in ameliorating the condition of CHD patients and reducing mortality, the prognosis for CHD patients following PCI remains disheartening (3, 4). Studies reveal that the incidence of cardiovascular endpoint events, such as postoperative myocardial infarction, revascularization, and all-cause mortality, ranges from 5% to 15% after PCI, posing a severe threat to patients' quality of life and health (5, 6).

Exercise emerges as a cornerstone in the secondary prevention of cardiovascular diseases, as it can slow and inhibit the progression of atherosclerosis, enhance cardiovascular and pulmonary health, stave off disease progression, and diminish the risk of adverse cardiovascular events (7). Unfortunately, data indicates that approximately 50%–87% of patients following cardiac surgery fail to engage in adequate exercise training, with over 70% experiencing some degree of kinesiophobia (8). Kinesiophobia is defined as an “irrational and excessive fear of physical activity due to heightened sensitivity to pain and concerns about secondary injuries that may impede recovery” (9). Compared to other ailments, CHD patients exhibit a higher prevalence of kinesiophobia, which not only diminishes their aerobic capacity and muscle strength but also heightens the risk of cardiac events and mortality, thereby compromising their quality of life (10, 11).

A myriad of factors contribute to the development of kinesiophobia in CHD patients, with adverse emotions and self-efficacy playing pivotal roles (12). On one hand, CHD patients bear a heavy burden due to symptoms stemming from the disease, deteriorating quality of life, and increased risks of death and hospitalization, leading to pervasive anxiety and depression among those diagnosed with CHD (13). Additionally, as interventions like PCI involve a certain degree of invasiveness, patients are more prone to anxiety and depression post-surgery, which in turn fuels worries about potential bodily mishaps during exercise, exacerbating their fear of movement (14). On the other hand, self-efficacy in CHD patients pertains to their belief in their ability to manage cardiovascular signs and symptoms (15). Given the patients' limited understanding of disease-related knowledge and lack of initiative in managing their condition, their self-efficacy often remains low, depriving them of adequate self-care knowledge and abilities. This fear that exercise might harm or re-injure their bodies inevitably propels the progression of kinesiophobia (16, 17).

Cardiac rehabilitation (CR) stands as a vital component of contemporary CHD care, considered a priority in countries with a high prevalence of CHD. CR encompasses medical evaluations, patient assessments, exercise regimens, behavioral counseling, risk factor management, patient education, and psychosocial support, all of which significantly enhance patients' health and improve their quality of life (18, 19). Exercise, a critical element within CR, plays a crucial role; however, the presence of kinesiophobia often triggers resistance in patients, hindering their compliance with CR interventions and severely undermining the efficacy of CR in CHD patients (20, 21). Research suggests that CR can effectively mitigate kinesiophobia in CHD patients, and integrating psychological interventions during CR significantly enhances the intervention's efficacy and patients' quality of life (22). Therefore, this study aims to incorporate psychological interventions into the CR process for CHD patients to explore the impact of psychologically-enhanced CR on kinesiophobia and provide insights to improve the quality of life and the efficacy of CR interventions for CHD patients.

## 2 Materials and methods

### 2.1 Patient information

This investigation encompassed patients diagnosed with CHD at Bethune International Peace Hospital between June 2022 and June 2023. The inclusion criteria for enrollment were as follows: (1) age ≤ 75 years; (2) confirmed diagnosis of CHD via coronary angiography and receipt of percutaneous coronary intervention (PCI) during hospitalization; (3) absence of physical or psychological impairments hindering mobility; (4) cardiac function classified as ≤III; (5) lucid consciousness, capable of providing accurate responses, and able to independently or with minimal assistance complete questionnaires. Exclusion criteria comprised: (1) presence of severe comorbidities affecting other organ systems; (2) individuals with motor dysfunction; (3) clinical prognoses indicating a life expectancy of less than 6 months; (4) refusal or inability to provide informed consent; (5) participation in another interventional clinical trial within 30 days preceding or concurrent with enrollment. This study received ethical approval from the Ethics Committee of Bethune International Peace Hospital (Approval No.: 2023-KY-95) and obtained written informed consent from all participants, in strict adherence to the principles of the Declaration of Helsinki and pertinent ethical guidelines.

### 2.2 Sample size calculation

In order to detect a moderate effect size (specifically, a standardized effect of at least 0.3 standard deviations) of psychological intervention on the primary outcome, with a statistical power of 0.80 and a significance level (*α*) set at 0.05, a total of 120 participants (60 in each group) were deemed necessary. Accounting for an anticipated 5% dropout rate, it was planned to recruit 127 participants. Nevertheless, due to the restricted number of patients enrolling in the CR program, the pre - determined sample size could not be achieved.

### 2.3 Randomization process

Participants were allocated to either the Conventional CR Group or the Psychological Intervention Group using a random number table following the subsequent procedure: Each participant was assigned a numerical identifier ranging from 01 to 120. To ascertain the group assignment for each individual, we initiated with the number located in the fourth column and third row of the random number table and subsequently obtained random numbers in a sequential manner along the same direction. These random numbers were then divided by the number of groups, which was two. The resultant remainder dictated each patient's group allocation. Specifically, if the remainder was zero, the patient was assigned to the Psychological Intervention Group; conversely, if the remainder was one, the patient was assigned to the Conventional CR Group. Throughout the process, no supplementary checks were carried out to guarantee baseline comparability between the groups.

### 2.4 Intervention methods

#### 2.4.1 Conventional CR group

Patients allocated to the conventional CR group underwent a standardized cardiac rehabilitation protocol, initiated within 24 h postoperatively. Specialized cardiac rehabilitation nurses conducted comprehensive evaluations, encompassing cardiac function, exercise tolerance, risk factors, and potential complications, to devise individualized rehabilitation plans integrating exercise, education, pharmacotherapy, and dietary management. In patients, the progression of exercise modalities begins with in - bed activities, transitions to bedside activities, followed by indoor activities, and ultimately culminates in outdoor activities. Considering the patients’ cardiac function and exercise tolerance, it is recommended that the peak exercise intensity be set at a Borg scale rating of 13, accompanied by an elevation of 20–30 beats per minute in the resting heart rate. This intensity should be dynamically adjusted over the course of the exercise program.Regarding exercise duration, each session commences at 10 min and is incremented by 5 min daily until a stable duration of 30–40 min per day is achieved. Patients are advised to engage in exercise 1–2 times daily, 5–7 days a week, for a consecutive period spanning 4–6 weeks. In the event that a patient does not attain the pre - specified exercise intensity within the allotted activity time on a particular day, the subsequent stage of the exercise regimen should be initiated on the following day (23, 24). Educational modules addressed the etiology, pathophysiology, clinical manifestations, therapeutic strategies, and prognostic outcomes of CHD, emphasizing the benefits and critical aspects of PCI, alongside guidance on medication adherence, risk factor mitigation, prevention of complications and recurrence, emergency response protocols, lifestyle modifications, work-rest equilibrium, emotional regulation, stress management, and enhancement of self-management competencies (25). Educational sessions were conducted for 20–30 minutes, once to twice weekly. Tailored to the individual circumstances of patients, medications including anti - platelet agents, anticoagulants, lipid - regulating and antihypertensive medications, as well as *β* - blockers, ought to be administered in strict accordance with medical prescriptions. Systematically assess the therapeutic efficacy and adverse events associated with these medications. Subsequently, make appropriate adjustments to the dosage and formulation of the drugs. Provide patients with comprehensive guidance on proper medication intake to preclude missed doses or overdosing, thereby enhancing medication adherence (26). Dietary interventions were customized based on the patient’s weight, lipid profile, and glycemic indices, advocating for a diet low in sodium, fat, and sugar, yet rich in fiber, while avoiding high-cholesterol, high-saturated fat, and high-purine foods. Total daily caloric and fluid intake were meticulously regulated, with an emphasis on incorporating fresh fruits and vegetables to sustain optimal weight and nutritional balance (27).

#### 2.4.2 Psychological intervention group

In the psychological intervention group, patients participated in a cardiac rehabilitation program integrated with psychological components, commencing within 24 h post-surgery. Skilled psychiatric nurses conducted psychological evaluations, assessing both negative emotions, such as anxiety, depression, fear, and feelings of inferiority, alongside positive psychological attributes, including confidence, self-efficacy, and coping mechanisms. Tailored psychological care plans were devised based on these assessments, incorporating elements of psychological counseling, support, guidance, and training. The counseling embraced humanistic, cognitive-behavioral, and problem-solving methodologies to establish a bond of trust and respect, attentively listening to patients' psychological concerns, discerning their psychological state, analyzing their psychological challenges, and offering reasoned psychological advice to dispel misconceptions and fortify psychological resilience (28). Each counseling session ranged from 30 to 60 min, occurring once or twice weekly for a duration of 4 to 6 weeks. Psychological support was rendered through empathetic engagement, words of encouragement, commendation, and solace, offering patients profound understanding and concern, satisfying their psychological needs, boosting their self-confidence and self-esteem, and fostering positive emotions while mitigating psychological pressures (29). Support sessions lasted 10–20 min, conducted once or twice daily for 4–6 weeks. Psychological guidance utilized techniques such as relaxation, breathing exercises, meditation, music, and art therapy to assist patients in regulating their breathing, relaxing their muscles, and calming their minds, cultivating a state of mindful awareness and enabling the expression of inner sentiments through music and art, thus aiding in the alleviation of psychological burdens and the reduction of psychological tension (30). These guidance sessions lasted 20–40 min, two to three times weekly, over a period of 4–6 weeks. Psychological training encompassed methods such as self-suggestion, self-motivation, self-reward, and self-feedback, teaching patients to employ positive self-talk to enhance their sense of self-efficacy, encouraging them to set attainable goals, providing fitting rewards, and offering feedback on their progress and accomplishments, thereby fostering a sense of self-worth (31). Training sessions were 10–20 min in length, once or twice daily. In this particular group, the medications administered to the patients are prescribed and meticulously monitored by clinical psychiatrists, cardiologists, along with other duly qualified healthcare professionals. The cardiac rehabilitation training for the psychological intervention group paralleled those of the conventional cardiac rehabilitation group.

Owing to China's medical insurance policy, the length of hospitalization for all patients is restricted to a period of 4–6 weeks. Upon discharge from the hospital, patients are advised to persist with the exercise regimen as per the pre—established exercise training protocol.Subsequently, physicians and associated responsible nursing personnel will initiate follow - up interventions for the patients via home visits, telephone calls, and outpatient consultations. The scope of the follow - up encompasses a comprehensive assessment, including an in - depth understanding of the patients' medical conditions, a thorough analysis of their psychological states, a reiteration of health education regarding the disease, and the provision of guidance on medication usage, dietary habits, and physical exercise.

In the present study, the training courses for all patients were carried out on the same day. Both groups were subjected to interventions over a span of 2 months.

### 2.5 Observational indicators

#### 2.5.1 Adverse emotions

Prior to and subsequent to the intervention, the patient's adverse emotions were assessed utilizing the Self-Rating Depression Scale (SDS) and the Self-Rating Anxiety Scale (SAS). Both scales are self-administered, consisting of 20 items, each graded on a 1–4 scale. Higher scores indicate a more severe state of anxiety and depression in the patient (32).

#### 2.5.2 Self-efficacy

Pre- and post-intervention, the patient's self-efficacy was evaluated using the Multidimensional Exercise Self-Efficacy Scale (MSES). This scale encompasses three dimensions—coping efficacy, time management efficacy, and task efficacy—with a total of 9 items, each scored between 0 and 10. The score for each dimension is the sum of its constituent item scores, and the total scale score ranges from 0 to 90. Higher scores reflect a stronger sense of self-efficacy in the patient (33).

#### 2.5.3 Exercise phobia

Before and after the intervention, the patient's exercise-related fear was assessed using the TSK-Heart Scale, specifically tailored for cardiac patients. This scale includes four dimensions—dysfunction, exercise fear, risk perception, and exercise avoidance—with 17 items in total, each graded on a 4-point Likert scale. The score for each dimension is the sum of its item scores, with the total scale score ranging from 17 to 68. Higher scores indicate a heightened level of exercise-related fear in the patient (34).

#### 2.5.4 Quality of life

Pre- and post-intervention, the patient's quality of life was evaluated using the Short Form Health Survey (SF-36). This scale covers four dimensions—social functioning, physical functioning, mental health, and bodily pain—with 36 items in total, each scored between 0 and 100. The score for each dimension is the sum of its item scores, and the overall scale score is an average of all dimension scores. Higher scores signify a better quality of life for the patient (35).

### 2.6 Statistical methods

This study employed SPSS version 25.0 software for the analysis of all data. Quantitative data were expressed as mean ± standard deviation (x̅ ± sd). Between-group comparisons were conducted using an independent samples *t*-test, while within-group pre- and post-intervention comparisons were performed using a paired samples *t*-test. Qualitative data were presented as numbers/percentages (n/%), with comparisons made using the chi-square test. Statistical significance was established at a *P* value less than 0.05.

## 3 Results

### 3.1 Patient baseline information

**Figure 1:**
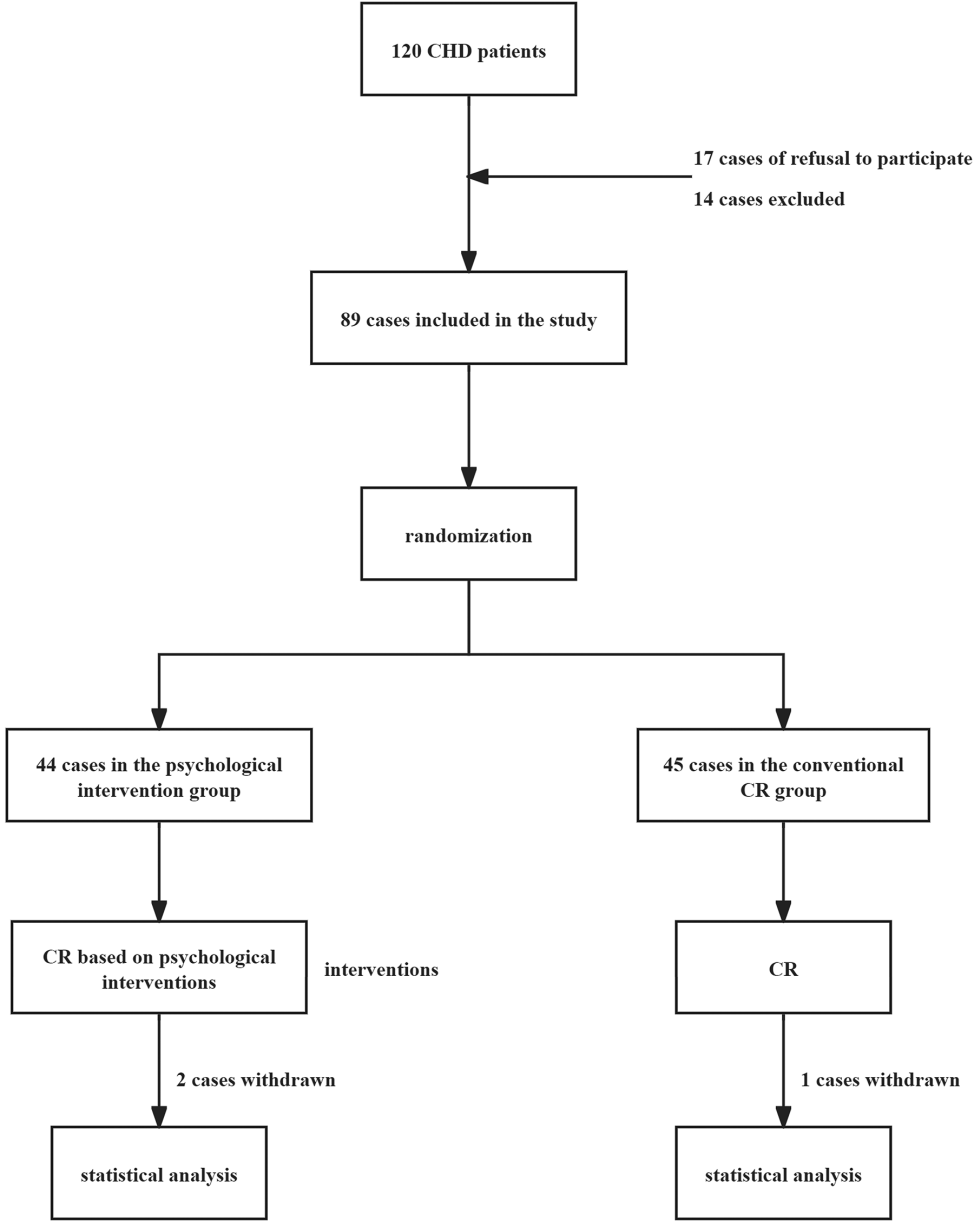
Experimental flowchart.

This investigation encompassed a cohort of 120 individuals diagnosed with coronary heart disease (CHD), who sought medical attention at Bethune International Peace Hospital between June 2022 and June 2023. Among these, 17 patients (14.17%) declined participation, while 14 patients (11.67%) were excluded based on predefined criteria. Consequently, 89 participants were enrolled and randomly allocated into two distinct groups: 44 individuals were assigned to the psychological intervention cohort, and 45 to the conventional cardiac rehabilitation (CR) cohort. During the course of the intervention, 2 participants from the psychological intervention group and 1 from the conventional CR group withdrew from the study. Ultimately, 86 patients successfully completed the trial, comprising 42 in the psychological intervention group and 44 in the conventional CR group. Comparative analysis revealed no statistically significant disparities in baseline characteristics, including gender, age, duration of illness, cardiac functional classification, number of affected vessels, or comorbid conditions, between the two groups (*P* > 0.05). (Table 1, Figure 1)

**Table 1:** Patient baseline information.

Parameter	Classification	Psychological intervention group (*n* = 42)	Conventional CR group (*n* = 44)	t/X^2^	*P*
Age (years):		70.19 ± 5.55	69.45 ± 4.94	0.6359	0.5150
Gender (male/female)		29/13	31/13	0.0202	0.8870
Duration of illness (years)		5.79 ± 1.02	5.89 ± 1.10	0.4366	0.6635
Heart function classification	Ⅰ	9	9	0.0168	0.9916
	Ⅱ	21	22		
	Ⅲ	12	13		
Number of affected vessels	1	18	17	0.1859	0.9112
	2	13	14		
	3	11	13		
Comorbidities	Hypertension	22	21	0.5771	0.7493
	Hyperlipidemia	14	14		
	Diabetes Mellitus	6	9		

### 3.2 Patient adverse emotional states

Comparative analysis revealed no statistically significant disparity in the SAS and SDS scores between the two patient cohorts prior to the intervention (*P* > 0.05). Post-intervention, both groups exhibited a marked reduction in SAS and SDS scores compared to their pre-intervention baselines (*P* < 0.05). Notably, the psychological intervention group demonstrated significantly lower SAS and SDS scores than the conventional CR group (*P* < 0.05) (Table 2).

**Table 2:** Patient adverse emotional states.

Group	SAS score		SDS score	
	Pre-intervention	Post-intervention	Pre-intervention	Post-intervention
Psychological intervention group (*n* = 42)	62.64 ± 1.14	25.71 ± 4.04*	62.55 ± 1.17	30.98 ± 4.22*
Conventional CR group (*n* = 44)	62.66 ± 1.16	27.95 ± 4.13*	62.50 ± 1.15	34.30 ± 4.13*
*t*	0.0806	2.5411	0.1998	3.6870
*P*	0.9360	0.0129	0.8421	0.0004

SAS, self-rating anxiety scale; SDS, self-rating depression scale.

* *P* < 0.05, indicative of statistical significance when compared to the pre-intervention phase within the same group.

### 3.3 Patient self-efficacy

Upon meticulous comparison, no statistically significant disparities were discerned in the MSES scores across various dimensions and the overall score between the two cohorts of patients prior to the implementation of the intervention (*P* > 0.05). Subsequent to the intervention, a notable enhancement in MSES scores across all dimensions and the aggregate score was observed in both patient groups when juxtaposed with their pre-intervention benchmarks (*P* < 0.05). Notably, the psychological intervention group exhibited substantially elevated MSES scores across dimensions and the total score, significantly surpassing those of the conventional CR group (*P* < 0.05). (Refer to Table 3 for detailed data.)

**Table 3:** Patient self-efficacy.

MSES score	Time	Psychological intervention group (*n* = 42)	Conventional CR group (*n* = 44)	*t*	*P*
Coping efficacy	Pre-intervention	4.45 ± 1.93	4.36 ± 2.40	0.1885	0.8509
	Post-intervention	7.71 ± 4.11*	5.77 ± 3.02*	2.5051	0.0142
Time management efficacy	Pre-intervention	16.26 ± 4.32	16.18 ± 4.89	0.0804	0.9361
	Post-intervention	22.83 ± 4.39*	19.14 ± 6.14*	3.2001	0.0019
Task efficacy	Pre-intervention	17.19 ± 6.25	16.57 ± 5.97	0.4723	0.6379
	Post-intervention	22.36 ± 4.25*	19.95 ± 6.16*	2.0943	0.0392
Total Score	Pre-intervention	37.90 ± 12.37	37.11 ± 13.04	0.2884	0.7737
	Post-intervention	52.90 ± 12.62*	44.86 ± 15.15*	2.6678	0.0092

MSES, multi-dimensional sport self-efficacy scale.

* *P* < 0.05, indicative of statistical significance when compared to the pre-intervention phase within the same group.

### 3.4 Levels of kinesiophobia in patients

Upon meticulous comparison, prior to the intervention, no statistically significant disparities were observed in the TSK-Heart subscale scores and total scores between the two cohorts of patients (*P* > 0.05). Subsequent to the intervention, both groups manifested a discernible diminution in TSK-Heart subscale scores and total scores relative to their pre-intervention baselines (*P* < 0.05). Furthermore, the psychological intervention cohort exhibited markedly lower TSK-Heart subscale scores and total scores in comparison to the conventional CR group (*P* < 0.05) (Table 4).

**Table 4:** Levels of kinesiophobia in patients.

TSK-Heart Score	Time	Psychological intervention group (*n* = 42)	Conventional CR group (*n* = 44)	*t*	*P*
Dysfunction	Pre-intervention	13.43 ± 1.36	13.30 ± 1.23	0.4755	0.6357
	Post-intervention	7.97 ± 1.14*	9.98 ± 1.11*	8.2594	<0.001
Exercise fear	Pre-intervention	15.81 ± 2.31	15.75 ± 2.46	0.1155	0.9083
	Post-intervention	10.24 ± 2.13*	12.59 ± 2.27*	4.6096	<0.001
Risk perception	Pre-intervention	13.38 ± 1.32	13.23 ± 1.38	0.5268	0.5997
	Post-intervention	7.86 ± 1.12*	9.34 ± 1.27*	5.7300	<0.001
Exercise avoidance	Pre-intervention	13.43 ± 1.23	13.15 ± 1.34	0.9741	0.3328
	Post-intervention	7.81 ± 1.17*	9.41 ± 1.23*	6.1753	<0.001
Total Score	Pre-intervention	56.05 ± 6.10	55.43 ± 6.25	0.4620	0.6453
	Post-intervention	33.88 ± 5.39*	41.32 ± 5.99*	6.0452	<0.001

TSK-Heart, exercise fear scale for patients with heart disease.

* *P* < 0.05, indicative of statistical significance when compared to the pre-intervention phase within the same group.

### 3.5 Quality of life in patients

A comparative analysis revealed no statistically significant disparities in the SF-36 domain scores and overall scores between the two patient cohorts prior to the intervention (*P* > 0.05). Following the intervention, both groups experienced marked enhancements in their SF-36 domain scores and total scores relative to their pre-intervention baselines (*P* < 0.05). Notably, the psychological intervention group exhibited superior scores across all SF-36 domains and in the aggregate score, significantly surpassing the conventional CR group (*P* < 0.05) (Table 5).

**Table 5:** Quality of life in patients.

SF-36 Score	Time	Psychological intervention group (*n* = 42)	Conventional CR group (*n* = 44)	*t*	*P*
Social functioning	Pre-intervention	72.53 ± 7.01	72.55 ± 7.14	0.0142	0.9887
	Post-intervention	85.02 ± 8.81*	79.14 ± 7.44*	3.35	0.0012
Physical functioning	Pre-intervention	71.52 ± 7.02	71.55 ± 6.95	0.0144	0.9885
	Post-intervention	85.02 ± 8.80*	78.14 ± 7.93*	3.8159	0.0003
Mental health	Pre-intervention	69.52 ± 5.89	69.41 ± 5.83	0.0907	0.9280
	Post-intervention	86.02 ± 7.48*	79.52 ± 7.07*	4.1433	0.0001
Bodily pain	Pre-intervention	70.62 ± 6.90	69.41 ± 6.84	0.8162	0.4167
	Post-intervention	85.64 ± 8.93*	80.05 ± 7.95*	3.0738	0.0029
Total Score	Pre-intervention	71.05 ± 6.65	70.73 ± 6.58	0.2246	0.8229
	Post-intervention	85.43 ± 8.45*	79.21 ± 7.54*	3.6048	0.0005

SF-36: The Concise Health Status Assessment Scale.

* *P* < 0.05, indicative of statistical significance when compared to the pre-intervention phase within the same group.

## 4 Discussion

Patients with CHD often exhibit heightened levels of exercise phobia, which markedly impairs their physical function and overall quality of life (36). CR, a multifaceted intervention designed to enhance patients' health status and quality of life, has demonstrated significant efficacy in clinical practice (37). This study integrates psychological intervention with CR, establishing a CR protocol rooted in psychological strategies. It was observed that CR augmented with psychological interventions effectively alleviated patients' negative emotions, bolstered their sense of self-efficacy, and ultimately diminished their levels of exercise phobia, thereby significantly improving their quality of life.

Negative emotions and low self-efficacy are primary drivers of exercise phobia in heart disease patients (38, 39). The findings of this study indicate that following CR interventions incorporating psychological strategies, patients' scores on the SAS, SDS, and all dimensions of the MSES and total scores showed marked improvement compared to those who underwent conventional CR alone. This underscores the pivotal role of psychological-based CR interventions in alleviating negative emotions and enhancing self-efficacy in heart disease patients, consistent with previous research by McKenzie et al. (40). One rationale for this is that during CR, exercise regimens positively influence patients, fostering stress management, attitudinal shifts, improved outlooks, self-reliance, confidence, trust, and a healthier mindset. These psychological transformations enable patients to confront the various pressures associated with their condition more positively and adopt effective coping strategies (41). Furthermore, during CR, patients' comprehension and mastery of disease-related and self-care knowledge deepen, and psychological improvements further amplify this effect, thereby strengthening their self-efficacy. Patients increasingly perceive their ability to apply this knowledge and skills in real-world settings and trust that these competencies will yield favorable health outcomes (42). Additionally, patients exhibit high receptivity to psychological interventions, and their preferences during CR are better understood through psychological approaches. This makes patients more inclined to communicate with family members or healthcare providers, thereby garnering greater social support and adopting a more positive stance toward their condition. Psychological interventions further mitigate the impact of negative emotions and low self-efficacy during CR, expediting the manifestation of its benefits in patients (43, 44).

The results also reveal that following CR interventions rooted in psychological strategies, patients' TSK-Heart scores across all dimensions and total scores, as well as SF-36 scores in all dimensions and total scores, showed significant improvement compared to those who received conventional CR alone. This suggests that psychological-based CR interventions exert a pronounced effect in reducing exercise phobia levels in heart disease patients and significantly enhancing their quality of life, aligning with previous research by Ploutarchou et al. (45). One explanation for this is that psychological-based CR interventions improve patients' negative emotions and self-efficacy, thereby eliminating or mitigating the root causes of exercise phobia. This empowers patients to engage more actively in CR interventions, enhancing their acceptance and adherence to CR regimens and facilitating the restoration of their physical functions (46, 47). Another factor is that during the process of receiving psychological-based CR interventions, patients' cognitive perspectives on exercise and their condition evolve, enabling them to directly confront the various impacts of exercise. Their motivation for recovery is heightened, and they develop a hopeful outlook for the future, which in turn fosters improvements in their quality of life (48).

It is important to recognize that this study has certain limitations. Firstly, constrained by the available conditions, this is a single - center study with a relatively small sample size. This limitation may, to some degree, compromise the representativeness and generalizability of the study findings. Therefore, additional multi - center, large - scale studies are required to validate the conclusions drawn from this research. Secondly, this study only enrolled patients with heart function classified as grade III or lower. The effectiveness of psychological interventions in CR for patients with higher grades of heart function remains to be further investigated. Thirdly, the intervention period in this study was relatively short, lasting only two months. Consequently, the long - term effects of psychological - based CR interventions on patients need further exploration and validation. Finally, in this study, only the questionnaire method was employed to evaluate the effectiveness of psychological interventions. Although the questionnaire method is a commonly used and effective approach for assessing the outcomes of psychological interventions, incorporating objective biological and clinical indicators in addition to the questionnaire survey may yield more robust results. Therefore, future research should adopt a multi - dimensional and comprehensive evaluation scheme, integrating objective biological and clinical indicators to comprehensively understand the impact of psychological interventions on patients with CHD.

In summary, implementing CR interventions grounded in psychological strategies during CR for heart disease patients can effectively mitigate their negative emotions, enhance their sense of self-efficacy, and ultimately diminish their levels of exercise phobia, thereby significantly improving their quality of life. Psychological-based CR interventions not only contribute to reducing patients' exercise phobia but also markedly enhance their quality of life, facilitating their successful reintegration into society and enabling them to lead healthier, more fulfilling lives.

## Data Availability

The original contributions presented in the study are included in the article/Supplementary Material, further inquiries can be directed to the corresponding author.

## References

[1] Timmis A Townsend N Gale CP Torbica A Lettino M Petersen SE European Society of Cardiology: cardiovascular disease statistics 2019. Eur Heart J. (2020) 41(1):12–85. 10.1093/eurheartj/ehz859 31820000

[2] Katta N Loethen T Lavie CJ Alpert MA. Obesity and coronary heart disease: epidemiology, pathology, and coronary artery imaging. Curr Probl Cardiol. (2021) 46(3):100655. 10.1016/j.cpcardiol.2020.100655 32843206

[3] Doenst T Thiele H Haasenritter J Wahlers T Massberg S Haverich A. The treatment of coronary artery disease. Dtsch Arztebl Int. (2022) 119(42):716–23. 10.3238/arztebl.m2022.0277 35912444 PMC9835700

[4] Tao S Tang X Yu L Li L Zhang G Zhang L Prognosis of coronary heart disease after percutaneous coronary intervention: a bibliometric analysis over the period 2004–2022. Eur J Med Res. (2023) 28(1):311. 10.1186/s40001-023-01220-5 37658418 PMC10472664

[5] Kook H Joo HJ Park JH Hong SJ Yu CW Lim D-S. A comparison between drug-eluting stent implantation and drug-coated balloon angioplasty in patients with left main bifurcation in-stent restenotic lesions. BMC Cardiovasc Disord. (2020) 20(1):83. 10.1186/s12872-020-01381-9 32070287 PMC7027103

[6] Lim SS Yang Y-L Chen S-C Wu C-H Huang S-S Chan WL Association of variability in uric acid and future clinical outcomes of patient with coronary artery disease undergoing percutaneous coronary intervention. Atherosclerosis. (2020) 297:40–6. 10.1016/j.atherosclerosis.2020.01.025 32062138

[7] Akyuz A. Exercise and coronary heart disease. Adv Exp Med Biol. (2020) 1228:169–79. 10.1007/978-981-15-1792-1_11 32342457

[8] Bäck M Cider Å Herlitz J Lundberg M Jansson B. Kinesiophobia mediates the influences on attendance at exercise-based cardiac rehabilitation in patients with coronary artery disease. Physiother Theory Pract. (2016) 32(8):571–80. 10.1080/09593985.2016.1229828 27726471

[9] Alpalhão V Cordeiro N Pezarat-Correia P. Kinesiophobia and fear avoidance in older adults: a scoping review on the state of research activity. J Aging Phys Act. (2022) 30(6):1075–84. 10.1123/japa.2021-0409 35303715

[10] Wang Z Zhang Y Wang Y Liu L Zhang J. Kinesiophobia and its associated factors in patients with coronary heart disease: a cross-sectional study based on latent feature analysis. BMJ Open. (2023) 13(7):e072170. 10.1136/bmjopen-2023-072170 37429691 PMC10335492

[11] Dąbek J Knapik A Gallert-Kopyto W Brzęk AMa Piotrkowicz J Gąsior Z. Fear of movement (kinesiophobia) - an underestimated problem in Polish patients at various stages of coronary artery disease. Ann Agric Environ Med. (2020) 27(1):56–60. 10.26444/aaem/106143 32208580

[12] Liu L Yang Q Li T Xie H Zeng B Zha L Prevalence and influencing factors of kinesiophobia in patients with heart disease: a meta-analysis and systematic review. Sci Rep. (2024) 14(1):18956. 10.1038/s41598-024-69929-9 39147837 PMC11327283

[13] Ski CF Taylor RS McGuigan K Long L Lambert JD Richards SH Psychological interventions for depression and anxiety in patients with coronary heart disease, heart failure or atrial fibrillation. Cochrane Database Syst Rev. (2024) 4(4):CD013508. 10.1002/14651858.CD013508.pub3 38577875 PMC10996021

[14] Rousseaux F Dardenne N Massion PB Ledoux D Bicego A Donneau A-F. Virtual reality and hypnosis for anxiety and pain management in intensive care units: a prospective randomised trial among cardiac surgery patients. Eur J Anaesthesiol. (2022) 39(1):58–66. 10.1097/EJA.0000000000001633 34783683 PMC8654253

[15] Nuraeni A Sugiharto F Anna A Sari E Mirwanti R Trisyani Y Self-efficacy in self-care and its related factors among patients with coronary heart disease in Indonesia: a rasch analysis. Vasc Health Risk Manag. (2023) 19:583–93. 10.2147/VHRM.S427488 37691747 PMC10492567

[16] Sugiharto F Nuraeni A Trisyani Y Putri AM Armansyah NA Zamroni AH. A scoping review of predictors associated with self-efficacy among patients with coronary heart disease. Vasc Health Risk Manag. (2023) 19:719–31. 10.2147/VHRM.S435288 37965056 PMC10642341

[17] Zhang S Wang Z Lin X Li Y Xue Y Ban J Kinesiophobia and self-management behaviour related to physical activity in Chinese patients with coronary heart disease: the mediating role of self-efficacy. Nurs Open. (2023) 10(1):105–14. 10.1002/nop2.1283 35773943 PMC9748108

[18] Dibben G Faulkner J Oldridge N Rees K Thompson DR Zwisler A-D Exercise-based cardiac rehabilitation for coronary heart disease. Cochrane Database Syst Rev. (2021) 11(11)):CD001800. 10.1002/14651858.CD001800.pub4 34741536 PMC8571912

[19] Su JJ Yu DS-F. Effects of a nurse-led eHealth cardiac rehabilitation programme on health outcomes of patients with coronary heart disease: a randomised controlled trial. Int J Nurs Stud. (2021) 122:104040. 10.1016/j.ijnurstu.2021.104040 34333211

[20] Sugiharto F Nuraeni A Trisyani Y Putri AM Armansyah NA. Barriers to participation in cardiac rehabilitation among patients with coronary heart disease after reperfusion therapy: a scoping review. Vasc Health Risk Manag. (2023) 19:557–70. 10.2147/VHRM.S425505 37671387 PMC10476659

[21] Bäck M Caldenius V Svensson L Lundberg M. Perceptions of kinesiophobia in relation to physical activity and exercise after myocardial infarction: a qualitative study. Phys Ther. (2020) 100(12):2110–9. 10.1093/ptj/pzaa159 32886775

[22] Baykal Şahin H Kalaycıoğlu E Şahin M. The effect of cardiac rehabilitation on kinesiophobia in patients with coronary artery disease. Turk J Phys Med Rehabil. (2021) 67(2):203–10. 10.5606/tftrd.2021.5164 34396071 PMC8343152

[23] Wang L Mei F Min M He X Luo L Ma Y. Adoption of the cardiopulmonary exercise test in the exercise ability and cardiopulmonary function rehabilitation of coronary artery disease (CAD) patients. BMC Cardiovasc Disord. (2024) 24(1):313. 10.1186/s12872-024-03958-0 38902630 PMC11191307

[24] Che Q Zhao L Huang B Li H. Effects of cardiac rehabilitation on symptoms and quality of life in cardiopulmonary exercise test-positive patients with non-obstructive coronary artery disease from high altitudes. Sichuan Da Xue Xue Bao Yi Xue Ban. (2024) 55(6):1507–14. 10.12182/20241160107 39990821 PMC11839350

[25] Spatola CAM Rapelli G Giusti EM Cattivelli R Goodwin CL Pietrabissa G Effects of a brief intervention based on acceptance and commitment therapy versus usual care for cardiac rehabilitation patients with coronary heart disease (ACTonHEART): a randomised controlled trial. BMJ Open. (2024) 14(6):e084070. 10.1136/bmjopen-2024-084070 38866567 PMC11177674

[26] Tan P Xie X Guo M Li M. Effects of rehabilitation management on lifestyle and quality of life of patients with coronary heart disease after percutaneous coronary intervention based on behavior change theory. Minerva Surg. (2023) 78(3):261–6. 10.23736/S2724-5691.22.09701-5 36117491

[27] Ma L-C Liu J Jiao C-L Du S-Y Zhang R-L Ding X-J Exploring the effects of an online learning platform in stage III cardiac rehabilitation for individuals with coronary heart disease: randomized controlled study. Medicine (Baltimore). (2024) 103(37):e39497. 10.1097/MD.0000000000039497 39287309 PMC11404953

[28] Li J Deng Y Jiang Y. The effectiveness of a web-based information-knowledge-attitude-practice continuous intervention on the psychological status, medical compliance, and quality of life of patients after coronary artery bypass grafting surgery: a parallel randomized clinical trial. J Cardiothorac Surg. (2024) 19(1):125. 10.1186/s13019-024-02618-w 38481263 PMC10935904

[29] Horn N Gärtner L Rastan AJ Andrási TB Lenz J Böning A Effects of a preoperative psychological expectation-focused intervention in patients undergoing valvular surgery—the randomized controlled ValvEx (valve patients’ expectations) study. Am Heart J. (2025) 282:156–69. 10.1016/j.ahj.2025.01.006 39827935

[30] Ya S-RT Lei Y-Y Bao L-X Cui X-S. Effects of nursing intervention based on a positive motivational model on cardiac function, self-management and quality of life in elderly patients with coronary heart disease. Eur Rev Med Pharmacol Sci. (2023) 27(17):7977–87. 10.26355/eurrev_202309_33557 37750626

[31] Cavalcante VN Mesquita ET Cavalcanti ACD Miranda JSDS Jardim PP Bandeira GMdS Impact of a stress reduction, meditation, and mindfulness program in patients with chronic heart failure: a randomized controlled trial. Arq Bras Cardiol. (2023) 120(10):e20220768. 10.36660/abc.20220768 37909602 PMC10586813

[32] Zheng Da Tan R-J Liu W Song P-C Li F-D. Sleep disturbances are associated with anxiety, depression, and decreased quality of life in patients with coronary heart disease. World J Psychiatry. (2023) 13(10):732–42. 10.5498/wjp.v13.i10.732 38058691 PMC10696286

[33] Yang Z Jia H Wang A. Predictors of home-based cardiac rehabilitation exercise adherence among patients with chronic heart failure: a theory-driven cross-sectional study. BMC Nurs. (2023) 22(1):415. 10.1186/s12912-023-01566-5 37926820 PMC10626687

[34] Qin J Xiong J Wang X Gao Y Gong K. Kinesiophobia and its association with fatigue in CHF patients. Clin Nurs Res. (2022) 31(7):1316–24. 10.1177/10547738221081230 35249417

[35] Nagyova I Jendrichovsky M Kucinsky R Lachytova M Rus V. Effects of nordic walking on cardiovascular performance and quality of life in coronary artery disease. Eur J Phys Rehabil Med. (2020) 56(5):616–24. 10.23736/S1973-9087.20.06120-1 32573523

[36] Çakal B Yıldırım M Emren SV. Kinesiophobia, physical performance, and health-related quality of life in patients with coronary artery disease. Postepy Kardiol Interwencyjnej. (2022) 18(3):246–54. 10.5114/aic.2022.122892 36751297 PMC9885221

[37] Taylor RS Dalal HM McDonagh STJ. The role of cardiac rehabilitation in improving cardiovascular outcomes. Nat Rev Cardiol. (2022) 19(3):180–94. 10.1038/s41569-021-00611-7 34531576 PMC8445013

[38] Zhang X Zhao Q Wang M Yang M Fan X. Fear of movement and its associated psychosocial factors in heart failure patients: a cross-sectional study. Eur J Cardiovasc Nurs. (2023) 22(3):273–81. 10.1093/eurjcn/zvac075 35989416

[39] Qin J Xiong J Chen C Wang X Gao Y Zhou Y Influencing factors of kinesiophobia in older patients with chronic heart failure: a structural equation model. Clin Cardiol. (2023) 46(7):729–36. 10.1002/clc.24024 37114367 PMC10352966

[40] McKenzie KM Park LK Lenze EJ Montgomery K Rashdi S Deych E A prospective cohort study of the impact of outpatient intensive cardiac rehabilitation on depression and cardiac self-efficacy. Am Heart J Plus. (2022) 13:100100. 10.1016/j.ahjo.2022.100100 36407054 PMC9671388

[41] Abdelbasset WK Alqahtani BA Elshehawy AA Tantawy SA Elnegamy TE Kamel DM. Examining the impacts of 12 weeks of low to moderate-intensity aerobic exercise on depression status in patients with systolic congestive heart failure—a randomized controlled study. Clinics (Sao Paulo). (2019) 74:e1017. 10.6061/clinics/2019/e1017 31576916 PMC6751366

[42] Heidari M Harandi PN Moghaddasi J Kheiri S Azhari A. Effect of home-based cardiac rehabilitation program on self-efficacy of patients with implantable cardioverter defibrillator. SAGE Open Nurs. (2023) 9:23779608231166473. 10.1177/23779608231166473 37124375 PMC10134157

[43] Liu Y Su M Lei Y Tian J Xue L Zhang L. Patient preferences for cardiac rehabilitation—a systematic review. Patient Prefer Adherence. (2023) 17:75–8. 10.2147/PPA.S392417 36636288 PMC9831083

[44] Wierenga KL Fresco DM Alder M Sattar A Moore SM. Preliminary efficacy of an emotion regulation intervention on physical activity and depressive and anxious symptoms in individuals in cardiac rehabilitation. J Cardiovasc Nurs. (2022) 37(3):296–305. 10.1097/JCN.0000000000000837 34321436 PMC8783925

[45] Ploutarchou G Savva C Karagiannis C Pavlou K O'Sullivan K Korakakis V. The effectiveness of cognitive behavioural therapy in chronic neck pain: a systematic review with meta-analysis. Cogn Behav Ther. (2023) 52(5):523–63. 10.1080/16506073.2023.2236296 37485605

[46] Öztürk İB Garip Y Sivas F Özden MP Bodur H. Kinesiophobia in rheumatoid arthritis patients: relationship with quadriceps muscle strength, fear of falling, functional status, disease activity, and quality of life. Arch Rheumatol. (2021) 36(3):427–34. 10.46497/ArchRheumatol.2021.8535 34870175 PMC8612492

[47] Brindisino F Garzonio F Giacomo GDI Pellegrino R Olds M Ristori D. Depression, fear of re-injury and kinesiophobia resulted in worse pain, quality of life, function and level of return to sport in patients with shoulder instability: a systematic review. J Sports Med Phys Fitness. (2023) 63(4):598–607. 10.23736/S0022-4707.22.14319-7 36305876

[48] Li L Sun Y Qin H Zhou J Yang X Li A A scientometric analysis and visualization of kinesiophobia research from 2002 to 2022: a review. Medicine (Baltimore). (2023) 102(44):e35872. 10.1097/MD.0000000000035872 37932995 PMC10627652

